# Diagnostic Models for Differentiating COVID-19-Related Acute Ischemic Stroke Using Machine Learning Methods

**DOI:** 10.3390/diagnostics14242802

**Published:** 2024-12-13

**Authors:** Eylem Gul Ates, Gokcen Coban, Jale Karakaya

**Affiliations:** 1 Institutional Big Data Management Coordination Office, Middle East Technical University, 06800 Ankara, Türkiye; 2 Department of Biostatistics, Hacettepe University, 06230 Ankara, Türkiye; jalekarakaya@gmail.com; 3 Department of Radiology, Hacettepe University, 06230 Ankara, Türkiye; drgokcencoban@gmail.com

**Keywords:** image processing, machine learning, COVID-19, long COVID-19, stroke, acute ischemia

## Abstract

**Backgrounds:** Although COVID-19 is primarily known as a respiratory disease, there is growing evidence of neurological complications, such as ischemic stroke, in infected individuals. This study aims to evaluate the impact of COVID-19 on acute ischemic stroke (AIS) using radiomic features extracted from brain MR images and machine learning methods. **Methods:** This retrospective study included MRI data from 57 patients diagnosed with AIS who presented to the Department of Radiology at Hacettepe University Hospital between March 2020 and September 2021. Patients were stratified into COVID-19-positive (*n* = 30) and COVID-19-negative (*n* = 27) groups based on PCR results. Radiomic features were extracted from brain MR images following image processing steps. Various feature selection algorithms were applied to identify the most relevant features, which were then used to train and evaluate machine learning classification models. Model performance was evaluated using a range of classification metrics, including measures of predictive accuracy and diagnostic reliability, with 95% confidence intervals provided to enhance reliability. **Results:** This study assessed the performance of dimensionality reduction and classification algorithms in distinguishing COVID-19-negative and COVID-19-positive cases using radiomics data from brain MR scans. Without feature selection, ANN achieved the highest AUC of 0.857 (95% CI: 0.806–0.900), demonstrating strong discriminative power. Using the Boruta method for feature selection, the k-NN classifier attained the best performance, with an AUC of 0.863 (95% CI: 0.816–0.904). LASSO-based feature selection showed comparable results across k-NN, RF, and ANN classifiers, while SVM exhibited excellent specificity and high PPV. The RFE method yielded the highest overall performance, with the k-NN classifier achieving an AUC of 0.882 (95% CI: 0.838–0.924) and an accuracy of 79.1% (95% CI: 73.6–83.8). Among the methods, RFE provided the most consistent results, with k-NN and the ANN identified as the most effective classifiers for COVID-19 detection. **Conclusions:** The proposed radiomics-based classification model effectively distinguishes AIS associated with COVID-19 from brain MRI. These findings demonstrate the potential of AI-driven diagnostic tools to identify high-risk patients, support optimized treatment strategies, and ultimately improve clinical implications.

## 1. Introduction

Severe acute respiratory syndrome coronavirus 2 (SARS-CoV-2) is a new coronavirus that causes viral pneumonia, which is known as coronavirus disease 2019 (COVID-19). It began in Wuhan, China, in late 2019 and spread quickly to become a global pandemic [1]. COVID-19, also referred to as a respiratory disease, damages blood vessels and other organs in addition to the lungs. Studies on the clinical traits, side effects, and prognosis of COVID-19-infected patients have been carried out since the pandemic started, with an emphasis on the virus’s neurological consequences. Intracerebral hemorrhage (ICH), cerebral venous thrombosis (CVT), and ischemic stroke (IS) are examples of common comorbidities [2].

Stroke is a serious public health concern, as it ranks as the second leading cause of death and morbidity globally [3]. Research indicates that early detection and treatment options, including intravenous thrombolysis and mechanical thrombectomy, significantly reduce morbidity and mortality. Nonetheless, there is still much to learn about the diagnosis and management of stroke linked to COVID-19 [4]. Several studies have demonstrated that, compared with traditional stroke treatment, the diagnosis and crucial early treatment of COVID-19-related stroke are delayed, which results in a marked increase in morbidity and death [4,5].

The SARS-CoV-2 virus primarily affects the respiratory system, but it also causes harm to other organs in the body. Tan et al. revealed that COVID-19 infection primarily causes severe pneumonia, with some patients developing neurological symptoms such as stroke. Furthermore, this study revealed that the incidence of stroke among COVID-19 patients ranges between 0.9% and 2.7% [6].

According to Asadi-Pooya and Simani’s study, 5% of patients with COVID-19 had a stroke, and the incidence of ischemic stroke was much greater than that of hemorrhagic stroke [7]. In a brief review study, Faroughi et al. examined 17 publications and reported that, because of the breakdown of fibrin, which results in increased blood coagulation, COVID-19 increases the risk of acute ischemic stroke, and D-dimer levels increase in patients with acute ischemic stroke after COVID-19 [8].

Liu et al. studied the transcriptome profiles of COVID-19 patients and ischemic stroke patients and discovered that ischemic stroke following COVID-19 could be linked to inflammatory pathways and the immune system [9]. Zhu et al. employed bioinformatics techniques to investigate probable pathogenic processes between ischemic stroke and COVID-19, as well as to suggest suitable medicines for treating COVID-19-related ischemic stroke [10].

In a review by Bahranifard et al., heterogeneous neuroradiological abnormalities caused by COVID-19 infection were examined, and it was found that ischemic stroke was the most common neuroradiological abnormality [11]. In a review by Liu et al. investigating the effects of COVID-19 and vaccines on stroke, it is suggested that the effects of COVID-19 on the brain and the mechanisms involved in increasing the risk of stroke should be investigated to better understand the pathophysiology of COVID-19-related stroke [12].

While these studies provide valuable insights, no research to date has utilized advanced radiomic features and machine learning (ML) techniques to specifically diagnose acute ischemic stroke associated with COVID-19. This represents a critical gap in the literature given the growing importance of AI-driven tools in medical imaging and diagnostics.

This study aims to bridge this gap by developing a diagnostic model to identify COVID-19-associated acute ischemic stroke using brain MR images. Leveraging advanced radiomic analysis and machine learning techniques, this study represents a pioneering effort to integrate computational tools for the diagnosis of neurological complications linked to COVID-19. By addressing this critical need, the proposed approach seeks to enhance diagnostic accuracy, facilitate early identification of high-risk patients, and inform clinical decision-making, thereby contributing to the broader understanding and management of COVID-19-related ischemic stroke.

## 2. Materials and Methods

### 2.1. Data Source, Patient Selection, and Image Acquisition

Patients who were diagnosed with acute ischemic stroke between March 2020 and September 2021 were scanned from the radiology database, and MRI data were obtained from the Picture Archiving and Communicating System (PACS) of our hospital. Among the initial 3175 patients aged 18–90 years with a prediagnosis of a cerebrovascular event (CVO), PCR records revealed that 3042 (95.8%) tested negative for COVID-19, while 133 (4.2%) tested positive. After applying the inclusion and exclusion criteria, 57 patients were retained for analysis, of which 30 (52.6%) were COVID-19 positive, representing a substantial subgroup for evaluating the impact of COVID-19 on cerebrovascular outcomes. The cranial MR images of 57 patients who were diagnosed with acute ischemia were re-evaluated by a neuroradiologist (GC) with 10 years of experience.

The inclusion criteria were as follows: (i) were >18 years old and <90 years old; (ii) had a PCR test result; (iii) had a brain MRI within 2 months after the PCR test (polymerase chain reaction); (iv) had diffusion MRI (b1000, ADC sequences); and (v) had a brain MRI including FLAIR, T2-weighted, susceptibility-weighted (SWI) sequences. Patients with and without COVID-19 were diagnosed based on a combination of PCR results, clinical evaluations, laboratory findings, and radiological assessments to ensure accurate classification.

The exclusion criteria for this study were as follows: (i) patients with a history of brain surgery or recurrent tumors were excluded to minimize the inclusion of confounding factors; (ii) MRI sequences demonstrating significant artifacts, including motion artifacts, phase encoding errors, wrapping artifacts, or pulsation artifacts, were excluded. In cases where such artifacts were confined to specific slices, only the affected slices were excluded to maintain overall image quality; (iii) cases of ischemic lesions with hemorrhagic transformation were excluded due to their potential to confound radiomic feature extraction; (iv) lesions with imaging features mimicking other pathologies were excluded, including neoplastic lesions, demyelinating diseases such as multiple sclerosis, vasculitic processes, infectious conditions including encephalitis, hematomas, and abscesses; and (v) individual MRI slices demonstrating microbleeds or focal susceptibility artifacts were excluded on a slice-by-slice basis to ensure the integrity of the radiomic analysis. These exclusion criteria were rigorously applied at both the patient and slice levels to ensure the clinical relevance and technical robustness of the dataset, reducing the risk of bias in the study outcomes. We also obtained patients’ demographic data (age at initial diagnosis and sex) from the Hacettepe University School of Medicine Hospital medical archive system. This study was approved by the Hacettepe University Medical and Health Sciences Research Committee (Ethics Committee number: GO 21/1012).

All scans were obtained on a 1.5 T MR imaging scanner (Aera; Siemens Healthcare, Erlangen, Germany) via a 20-channel phased array head coil. The conventional MRI protocol included axial FLAIR (TE/TR/TI: 78/7000/2220 ms, FOV: 230 × 185 mm; slice thickness [ST]/gaps: 5/2 mm; acquisition time [AT]: 4:06 min), axial 3D T1W MPRAGE (TE/TR/: 3/1680 ms, FOV: 240 × 195 mm; ST/gaps: 1.5/0.75 mm gaps; AT: 5:08 min), axial T2W turbo SE (TE/TR/: 199/3240 ms, FOV: 230 × 185 mm; ST/gaps: 5/2 mm; AT: 3:42 min), and DWI (TR/TE: 3400–5674/75–94 ms, FOV: 230 × 185 mm; ST/gaps: 5/1 mm; AT: 45 s).

**Figure 1:**
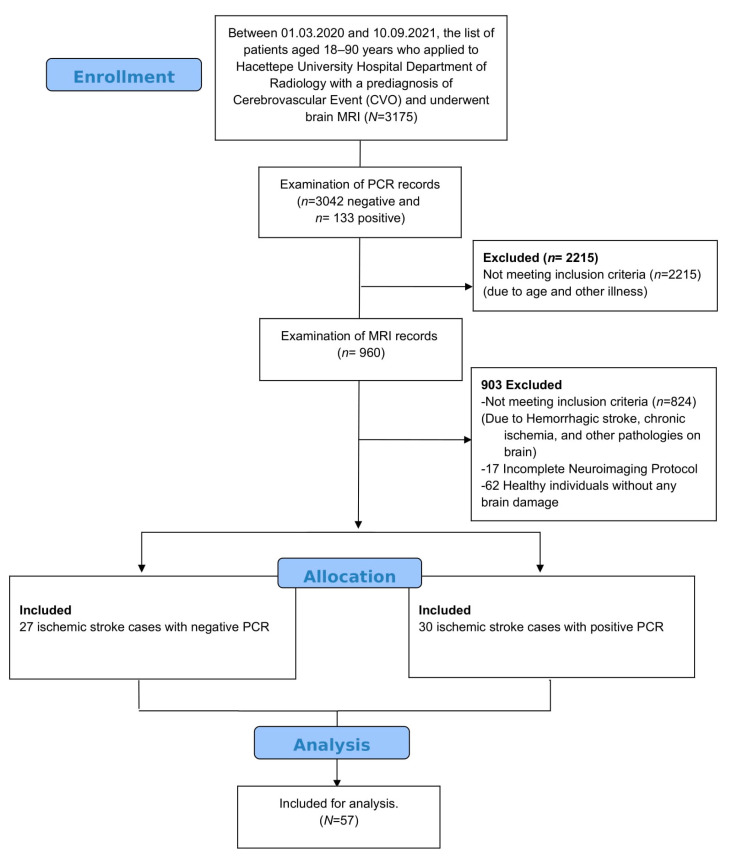
CONSORT diagram of patient screening.

Based on the DWI results, 57 patients (30 patients diagnosed with COVID-19 and 27 patients without COVID-19) were included in this retrospective study (Figure 1).

### 2.2. Proposed Methodology

**Figure 2:**
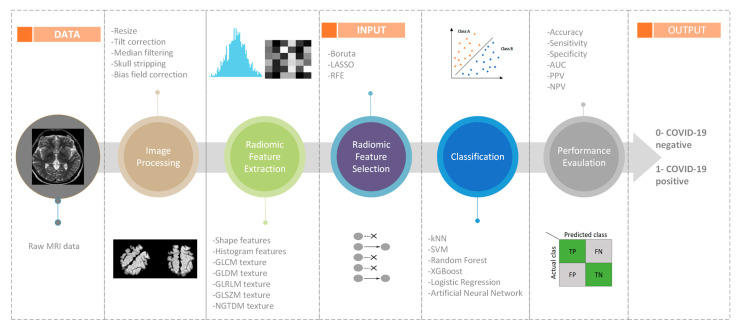
Flow diagram of the proposed method for detecting COVID-19 from DICOM images.

This paper focuses on COVID-19 associated with AIS and investigates the detection of such strokes via image processing, radiomics, and machine learning methods. Figure 2 presents a flowchart illustrating the proposed methodology, which involves steps such as image processing, radiomic feature extraction, radiomic feature selection, classification using machine learning algorithms, and the performance evaluation of the classification models. All the experimental stages were conducted using Python v3.10.0 software. STARD (Standards for Reporting of Diagnostic Accuracy Studies) checklist. The design and reporting of this study adhere to the STARD guidelines [13]

#### 2.2.1. Image Preprocessing

**Figure 3:**
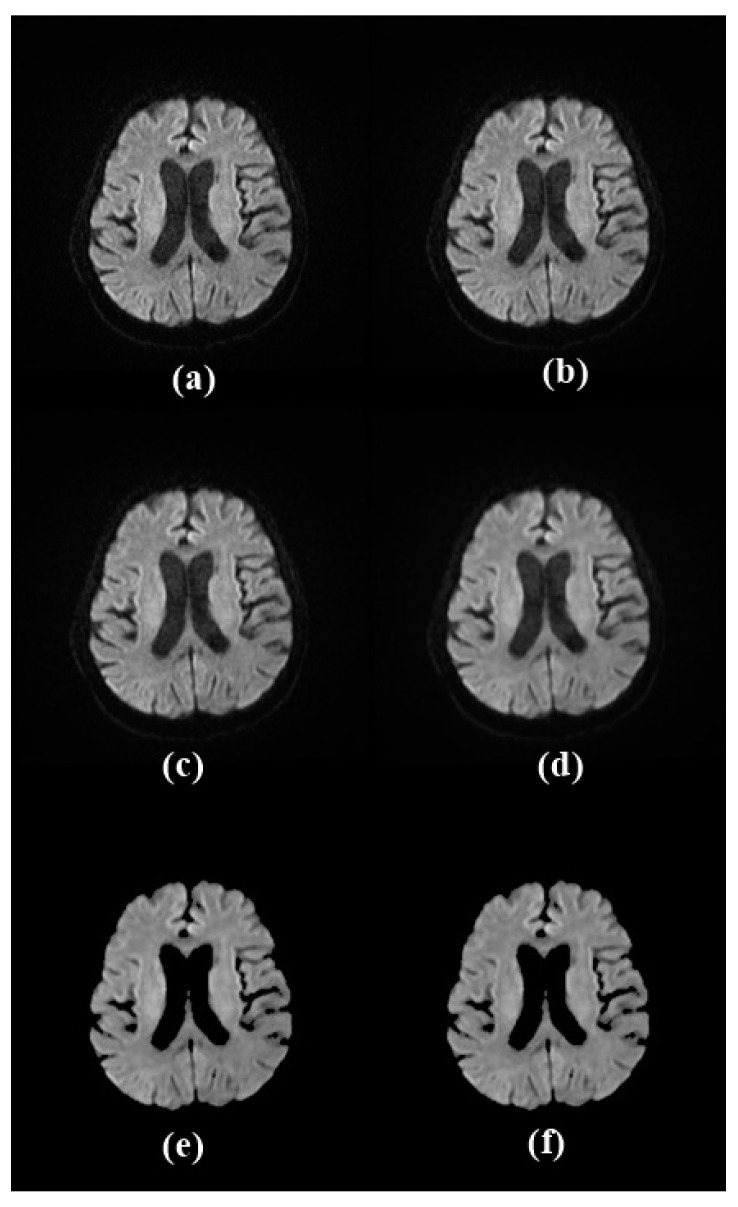
Visualization of image processing steps for the image of a COVID-19-positive patient: (**a**) original image, (**b**) resized image, (**c**) tilt corrected image, (**d**) noise removed image, (**e**) skull stripped image, (**f**) bias field corrected image.

During the initial stage of image processing, high-resolution MR images were resized to 256 × 256 using the resize function to reduce processing time and computational costs. Additionally, tilt correction was applied to address issues caused by patient movements during image recording, resulting in more accurate and smoother images [14]. Median filtering was applied to remove the noise generated during image recording. The median filter is preferred because it is less sensitive to extreme values and removes outliers by minimizing the loss of sharpness in the image. With this method, a more homogeneous background is obtained; therefore, abnormalities become more prominent [15]. Skull stripping, also known as brain segmentation, which is a preprocessing method proposed in brain MR imaging studies, is the process of removing non-brain regions from an image [16]. Finally, bias field correction was performed. Bias field correction adjusts image contrast affected by magnetic field variations. This correction depends on the magnetic field strength and becomes more pronounced at higher field strengths, such as 1.5 T, 3 T, or above. This can potentially influence MRI analysis [17]. Figure 3 clearly illustrates the preparation procedures, with all the applied processing approaches visually presented on an example image.

#### 2.2.2. Radiomics

The process of obtaining high-dimensional data from image features is called radiomics, which involves the mathematical description of medical images. Radiomics can be combined with clinical and genetic biomarkers to provide important information for diagnosing diseases, determining treatment options, and evaluating disease prognosis [18].

After image preprocessing, radiomic features were extracted using the PyRadiomics library. During this process, PyRadiomics applied standardization to normalize voxel intensities and spatial resolutions and performed intensity discretization to enhance feature robustness and reproducibility. These steps ensured the extracted features were consistent and suitable for statistical and predictive analysis.

In this study, a total of 95 radiomic features were extracted using shape features: first-order histogram, gray-level co-occurrence matrix (GLCM), gray-level difference matrix (GLDM), gray-level run length matrix (GLRLM), gray-level size zone matrix (GLSZM), and neighborhood gray-tone difference matrix (NGTDM) methods (Supplementary Table S1). To select the features associated with COVID-19 and eliminate irrelevant radiomic features, recursive feature elimination (RFE), least absolute shrinkage and selection operator (LASSO), and Boruta feature selection methods were utilized.

#### 2.2.3. Model Training and Performance Evaluation

In this study, a range of commonly used classifiers in the field of radiomics were employed, including k-nearest neighbor (k-NN), support vector machine (SVM), random forest (RF), extreme gradient boosting (XGBoost), logistic regression (LR), and artificial neural network (ANN) [19,20]. The selected radiomic features were input into ML algorithms for COVID-19 classification. The dataset was randomly split into 80% training and 20% testing sets using the shuffle function, ensuring that this split was performed on a patient-level basis to prevent data leakage between the sets. Evaluating model performance without hyperparameterization can lead to overfitting of the model to the data, especially in small and imbalanced datasets, and thus reduce the prediction success. Therefore, a tenfold cross-validation method was employed to evaluate the classifiers and enhance their generalizability while mitigating the risk of overfitting [21].

Hyperparameter tuning was conducted using the GridSearchCV method, which also utilized a 10-fold cross-validation process. GridSearchCV systematically evaluated each combination of hyperparameters and identified the optimal set, which was subsequently used for training the final model (Supplementary Table S2). To ensure robust and reliable results, each performance metric, including the area under the curve (AUC) from the receiver operating characteristic (ROC) curve, accuracy, sensitivity, specificity, positive predictive value (PPV), and negative predictive value (NPV), was calculated with 95% confidence intervals using 1000 bootstrap samples. In addition, no missing values were observed in the radiomic dataset.

The confusion matrix, also known as the error matrix, comprises four elements: true positive (TP), true negative (TN), false positive (FP), and false negative (FN). The performance metrics are generated as follows [22]:

Accuracy=TN+TP/(TP+FN+FP+TN)

Sensitivity=TP/(TP+FN)

Specificity=TN/(TN+FP)

PPV=TP/(TP+FP)

NPV=TN/(TN+FN)

The AUC is a useful metric for summarizing the overall diagnostic accuracy of a test and ranges from 0 to 1. Generally, an AUC of 0.5 means that the test cannot distinguish between patients with and without the disease or condition: 0.7 to 0.8 is considered acceptable, 0.8 to 0.9 is considered excellent, and more than 0.9 is considered outstanding [23].

## 3. Results

A total of 1273 images from 57 patients (of whom 616 were COVID-19 negative and 557 were COVID-19 positive) were used in this study. Among those in the COVID-19-negative group, 66.7% were women (*n* = 18), while 30% of those in the COVID-19-positive group were women. The average age of the patients was 60.88 ± 16.24 years, with a median of 62 years (range 22–88). The average age of those in the COVID-19-negative group was 53.85 ± 17.66 years, with a median of 56 years (range 22–81), and the average age of those in the COVID-positive group was 67.20 ± 11.93 years, with a median of 65 years (range 46–88). Detailed descriptive statistics of the patient demographics and groups are presented in Table 1.

Radiomics data extracted from patients’ brain MRI scans were identified as COVID-19-negative or COVID-19-positive using a variety of dimensionality reduction and classification algorithms. In Table 2, all the radiomic data were fed into machine learning algorithms without using feature selection. The accuracy values indicated that the RF, XGBoost, and ANN algorithms demonstrated similar performance. In terms of the AUC values, the algorithm that best discriminated between COVID-19-negative and COVID-positive patients was the ANN, which had an AUC value of 0.857 (95% CI: 0.806–0.900). Table 3 shows the classification results obtained using the Boruta method for radiomic feature selection. The Boruta algorithm revealed 25 radiomic characteristics that are useful for detecting COVID-19. Using this method, the highest performance was achieved with the k-NN algorithm, with an AUC of 0.863 (95% CI: 0.816–0.904).

Using LASSO for feature selection, 17 radiomics were chosen, as shown in Table 4. Considering accuracy and AUC metrics, the k-NN, RF, and ANN algorithms demonstrated good and comparable performance, while the SVM method stands out with its excellent specificity and high PPV scores. Table 5 shows the feature selection performance of classifiers that use the RFE technique. Forty-seven radiomic characteristics were found to be relevant by the RFE method for COVID-19 detection. The logistic regression classifier had the lowest accuracy value at 66.4% (95% CI: 60.4–72.3) and an AUC of 0.705 (95% CI: 0.637–0.772), while the k-NN classifier achieved the best accuracy value at 79.1% (95% CI: 73.6–83.8) and an AUC of 0.882 (95% CI: 0.838–0.924).

**Figure 4:**
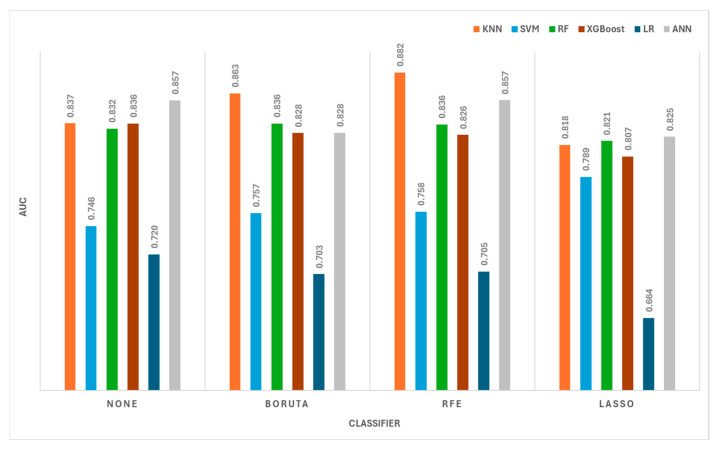
Comparison of the performance of different feature selection methods and classification methods for COVID-19 detection based on AUC values.

Figure 4 illustrates the classifiers’ discriminative performance under various feature selection techniques. Among these, the ANN and k-NN algorithms demonstrated the best performance in detecting COVID-19. LASSO produced the lowest performance results among the dimensionality reduction techniques, whereas the RFE method stood out by achieving the highest AUC value (0.882) with the k-NN algorithm. Furthermore, the RFE method demonstrated competitive and reliable performance across other classifiers.

## 4. Discussion

There is still uncertainty about the relationship between stroke and COVID-19. Previous studies have been limited in their ability to describe the characteristics of COVID-19-related acute ischemic brain MRI findings with conventional imaging methods or to conclude that conventional brain MRI findings are similar [24,25,26]. However, despite overlapping radiological findings, different etiopathogeneses imply that treatment options may vary [27]. As highlighted in Glavin et al., distinguishing between strokes and stroke mimics in COVID-19 patients is particularly challenging, emphasizing the importance of rapid and accurate diagnosis [28]. Our study demonstrated that COVID-19-positive patients and COVID-19-negative acute ischemic patients can be distinguished using classical machine learning methods with high accuracy and reliability.

In the clinical setting, the diagnosis of acute ischemic stroke is established by radiologists using the gold standard diffusion MRI sequence. However, distinguishing between stroke associated with COVID-19 and stroke due to chronic atherosclerotic disease in the same age group using imaging techniques is challenging. For instance, ischemia due to a primary vascular occlusion without an infectious disease and ischemia with an underlying viral infection, such as COVID-19, may both present as lesions with diffusion restriction on DWI-sequence and demonstrate hypo-intensity on T1WI, hyperintensity on T2WI, and no enhancement on post-contrast T1WI. These overlapping findings can complicate diagnosis and delay treatment decisions. This distinction is crucial because the treatment options differ, and it is important to differentiate between these two etiologies, which present with similar radiological images, to ensure appropriate treatment. According to the WHO definition, the detection of SARS-CoV-2 in respiratory or other non-central nervous system samples, combined with the exclusion of other potential causes, is sufficient for diagnosing COVID-19-related central nervous system complications [29]. Our study is one of the first to demonstrate the effectiveness of classical machine learning algorithms in distinguishing between COVID-19-associated acute ischemic strokes and strokes related to other causes based on radiological features.

Studies on artificial intelligence (AI) that have already been published in the literature have focused on using MR images to identify ischemia. Research has shown that the classification accuracy of deep learning models ranges from 88% to 97%, and that of classical machine learning techniques ranges from 89% to 93% [30,31,32,33,34]. However, there are currently no studies in the literature that specifically address the radiological imaging-based detection of COVID-19-related acute ischemic stroke. Our study is the first of its kind and has demonstrated excellent performance in classifying algorithms utilizing the hybrid methods we propose, particularly in identifying strokes associated with COVID-19.

In a study conducted to determine the effects of SARS-CoV-2 infection on ischemic stroke, clinical data from 205 ischemic stroke patients who were COVID-19-negative and 43 ischemic stroke patients who were COVID-19-positive were evaluated. Ischemic stroke patients exhibit similar clinical characteristics in both COVID-19-positive and COVID-19-negative patients [35]. However, the results of this study, which used brain MRI data from two groups of patients, revealed differences in the radiological images of those who tested positive for COVID-19 and those who tested negative when evaluated using classical machine learning methods.

COVID-19 is associated with a variety of mental and neurological symptoms, including anxiety, depression, headaches, and seizures [36,37]. In the United States, over 80% of hospitalized COVID-19 patients experienced neurological symptoms, which were linked to a fourfold increase in the risk of severe COVID-19 [38]. Individuals with preexisting mental or neurological conditions, such as dementia, depression, or psychosis, face higher mortality rates and worse outcomes when infected with SARS-CoV-2 [36,37]. Additionally, COVID-19 has been linked to a rise in acute cerebrovascular diseases [39] and a 42% increase in long-term neurological sequelae, including cognitive, memory, movement, and sensory disorders [40]. Poor outcomes in patients with COVID-19 and stroke may be related to the severity of the underlying medical condition, increased inflammation, or delays in acute stroke treatments such as thrombolysis or mechanical thrombectomy. Despite longer door-to-needle times, no significant delays were found in door-to-groin times for patients undergoing mechanical thrombectomy. Although the issue of a poor outcome would be the need for an appropriate combination of medical therapies, the results of our study suggest that the application of a machine learning approach could help determine the etiology of stroke, thereby enabling an appropriate combination of antiviral, corticosteroid, and anticoagulant therapies to be initiated while awaiting laboratory confirmation, potentially enhancing the chances of full recovery.

Acute ischemic stroke can become more severe and occur earlier in life due to various risk factors linked to SARS-CoV-2 and the emergence of COVID-19. These factors include endothelial cell damage, which causes increased inflammation and thrombosis, dysregulation of the renin‒angiotensin‒aldosterone system, hypoxemia related to cardiorespiratory failure, generalized hypercoagulability, a dysregulated immune response leading to cytokine release syndrome, and direct cytotoxic effects on the nervous system due to angiotensin-converting enzyme-2 (ACE-2) receptor uptake of the SARS-CoV-2 virus [41].

The hypothesized mechanisms for neurological injury and cerebrovascular events in patients with severe COVID-19 include cytokine storms, embolic events due to myocarditis and arrhythmia, hypoxia-induced ischemia and apoptosis, thrombotic microangiopathy, coagulopathy and thrombocytopenia, and direct viral invasion [42]. Similarly, acute venous thromboembolism, such as acute stroke, is classified as a critical condition in the COVID-19 severity classification [27]. During the period until diagnosis, disease progression can occur rapidly in stable patients, and viral loads are highest early in the infection course. Therefore, rapid initiation of therapy in high-risk populations (patients who are hospitalized or outpatients at high risk of complications) is rational and should be considered.

Recent news reports have indicated that AstraZeneca officially acknowledged that its COVID-19 vaccine, Covishield, can, in rare cases, trigger thrombosis with thrombocytopenia syndrome (TTS) [43,44]. However, considering the available data and the complex pathophysiology of COVID-19, it is speculated that the side effects of TTS may arise not directly from the vaccine but rather from the COVID-19 infection itself. The rapid development and deployment of vaccines during the pandemic were crucial in slowing the spread of the virus and reducing mortality. Therefore, it is likely that some rare side effects may be the result of immunological and hematological responses triggered by COVID-19 infection rather than the vaccine itself. As highlighted by Wong et al. and Sharif et al., the management of COVID-19-associated central nervous system complications (e.g., encephalitis, vasculitis, or encephalopathies) often requires combination therapy, including intravenous immunoglobulin, corticosteroids, and anticoagulants [45,46]. Therefore, making a rapid and accurate differential diagnosis of strokes related to COVID-19 is critical for initiating appropriate treatment. Our study suggests that the application of machine learning methods could support the differentiation of stroke etiologies by leveraging subtle differences in imaging features, thus enabling earlier and more appropriate interventions.

## 5. Limitations of the Study

The first limitation is related to the sample size. The deep learning algorithms used in medical imaging typically require large datasets, which are not easily accessible. Owing to the strict inclusion criteria in our study, we were unable to achieve a large sample size. Consequently, we could not employ deep learning models and instead utilized only machine learning algorithms. Another limitation is that all patients were collected from a single center. Although these patients reflect the general characteristics of acute stroke patients, the parameters used for the machine learning algorithms were optimized and determined specifically for this unique population. This indicates that the results of the study should be interpreted with caution. To test the generalizability of the study’s findings, multicenter studies involving different patient populations should be conducted.

## 6. Conclusions

This study presents a method for differentiating between COVID-19-related ischemia and classical acute ischemic strokes using traditional machine learning techniques. Although the advent of the COVID-19 pandemic caught healthcare providers unprepared, it is well established that other viral infections, including influenza and herpesviruses, also cause AIS [47]. While our findings specifically demonstrate that radiomic features derived from brain MRI can effectively differentiate COVID-19-related strokes, we believe this approach can provide a foundation for future research aimed at predicting ischemia caused by other viral infections. Our classifier achieved high accuracy in identifying high-risk patients requiring tailored treatment and follow-up, improving our understanding of the neurological effects of COVID-19 and supporting clinical and public health initiatives. 

## Figures and Tables

**Table 1:** Descriptive statistics.

Descriptives	Acute Ischemic Stroke Patients		Total
	COVİD-19-Negative	COVİD-19-Positive	
*n*	27	30	57
Age	53.85 ± 17.66	67.20 ± 11.93	60.88 ± 16.24
Sex			
Female	18 (66.7%)	9 (30.0%)	27 (47.4%)
Male	9 (33.3%)	21 (70.0%)	30 (52.6%)

**Table 2:** Performance of classification algorithms on the test set without feature selection (number of radiomic features = 95).

Classifier	Accuracy	Sensitivity	Specificity	AUC	PPV	NPV
k-NN (95% CI)	0.706 (0.647–0.762)	0.633 (0.540–0.723)	0.770 (0.693–0.846)	0.837 (0.786–0.883)	0.704 (0.602–0.800)	0.708 (0.628–0.784)
SVM (95% CI)	0.723 (0.660–0.775)	0.596 (0.505–0.688)	0.833 (0.758–0.892)	0.746 (0.679–0.808)	0.756 (0.667–0.846)	0.705 (0.628–0.775)
RF (95% CI)	0.749 (0.694–0.804)	0.688 (0.598–0.769)	0.802 (0.733–0.867)	0.832 (0.777–0.878)	0.750 (0.667–0.833)	0.748 (0.677–0.815)
XGBoost (95% CI)	0.766 (0.711–0.817)	0.679 (0.587–0.771)	0.841 (0.777–0.904)	0.836 (0.783–0.883)	0.787 (0.708–0.860)	0.752 (0.679–0.818)
LR (95% CI)	0.630 (0.566–0.694)	0.560 (0.469–0.647)	0.690 (0.613–0.768)	0.720 (0.653–0.781)	0.610 (0.505–0.705)	0.644 (0.557–0.724)
ANN (95% CI)	0.749 (0.694–0.809)	0.679 (0.590–0.764)	0.810 (0.738–0.876)	0.857 (0.806–0.900)	0.755 (0.667–0.835)	0.745 (0.669–0.815)

PPV: positive predictive value, NPV: negative predictive value, AUC: area under the curve, k-NN: k-nearest neighbor algorithm, SVM: support vector machine, RF: random forest, XGBoost: extreme gradient boosting, LR: logistic regression, ANN: artificial neural network, CI: confidence interval.

**Table 3:** Performance of classification models with feature selection based on the Boruta algorithm (number of radiomic features = 25).

Classifier	Accuracy	Sensitivity	Specificity	AUC	PPV	NPV
k-NN (95% CI)	0.753 (0.698–0.804)	0.697 (0.609–0.777)	0.802 (0.732–0.867)	0.863 (0.816–0.904)	0.752 (0.661–0.829)	0.754 (0.678–0.821)
SVM (95% CI)	0.728 (0.672–0.783)	0.615 (0.524–0.704)	0.825 (0.754–0.890)	0.757 (0.692–0.822)	0.753 (0.663–0.841)	0.712 (0.632–0.782)
RF (95% CI)	0.736 (0.681–0.796)	0.679 (0.589–0.766)	0.786 (0.708–0.855)	0.836 (0.788–0.885)	0.733 (0.646–0.819)	0.739 (0.661–0.809)
XGBoost (95% CI)	0.732 (0.677–0.787)	0.651 (0.563–0.750)	0.802 (0.726–0.869)	0.828 (0.776–0.879)	0.740 (0.652–0.824)	0.727 (0.650–0.800)
LR (95% CI)	0.694 (0.634–0.753)	0.606 (0.518–0.695)	0.770 (0.694–0.845)	0.703 (0.635–0.763)	0.695 (0.598–0.779)	0.693 (0.617–0.767)
ANN (95% CI)	0.728 (0.668–0.783)	0.651 (0.565–0.739)	0.794 (0.717–0.861)	0.828 (0.774–0.877)	0.732 (0.645–0.815)	0.725 (0.651–0.800)

**Table 4:** Performance of classification models with feature selection based on the LASSO algorithm (number of radiomic features = 17).

Classifier	Accuracy	Sensitivity	Specificity	AUC	PPV	NPV
k-NN (95% CI)	0.757 (0.706–0.813)	0.697 (0.611–0.777)	0.810 (0.734–0.875)	0.818 (0.760–0.873)	0.760 (0.670–0.841)	0.756 (0.681–0.823)
SVM (95% CI)	0.736 (0.681–0.791)	0.569 (0.469–0.672)	0.881 (0.821–0.934)	0.789 (0.729–0.846)	0.805 (0.711–0.892)	0.703 (0.632–0.778)
RF (95% CI)	0.740 (0.689–0.800)	0.633 (0.541–0.722)	0.833 (0.766–0.901)	0.821 (0.766–0.874)	0.767 (0.683–0.845)	0.724 (0.647–0.795)
XGBoost (95% CI)	0.728 (0.668–0.787)	0.615 (0.525–0.701)	0.825 (0.752–0.893)	0.807 (0.750–0.860)	0.753 (0.660–0.843)	0.712 (0.634–0.786)
LR (95% CI)	0.664 (0.600–0.719)	0.514 (0.421–0.611)	0.794 (0.720–0.862)	0.664 (0.590–0.727)	0.683 (0.584–0.784)	0.654 (0.573–0.725)
ANN (95% CI)	0.745 (0.689–0.804)	0.651 (0.563–0.734)	0.825 (0.753–0.889)	0.825 (0.767–0.879)	0.763 (0.682–0.848)	0.732 (0.655–0.803)

**Table 5:** Performance of classification models with feature selection based on the RFE algorithm (number of radiomic features = 47).

Classifier	Accuracy	Sensitivity	Specificity	AUC	PPV	NPV
k-NN (95% CI)	0.791 (0.736–0.838)	0.752 (0.667–0.832)	0.825 (0.761–0.891)	0.882 (0.838–0.924)	0.788 (0.707–0.872)	0.794 (0.723–0.863)
SVM (95% CI)	0.715 (0.660–0.774)	0.596 (0.500–0.689)	0.817 (0.752–0.884)	0.758 (0.698–0.818)	0.739 (0.646–0.826)	0.701 (0.626–0.771)
RF (95% CI)	0.745 (0.685–0.800)	0.670 (0.577–0.753)	0.810 (0.742–0.877)	0.836 (0.780–0.883)	0.753 (0.667–0.833)	0.739 (0.664–0.809)
XGBoost (95% CI)	0.753 (0.698–0.804)	0.688 (0.596–0.769)	0.810 (0.739–0.872)	0.826 (0.768–0.875)	0.758 (0.670–0.843)	0.750 (0.678–0.821)
LR (95% CI)	0.664 (0.604–0.723)	0.624 (0.532–0.721)	0.698 (0.617–0.783)	0.705 (0.637–0.772)	0.642 (0.543–0.731)	0.682 (0.603–0.760)
ANN (95% CI)	0.770 (0.719–0.821)	0.688 (0.596–0.775)	0.841 (0.774–0.902)	0.857 (0.805–0.899)	0.789 (0.707–0.865)	0.757 (0.686–0.828)

## Data Availability

The data presented in this study are available on reasonable request from the corresponding author due to ethical restrictions.

## Supplementary Materials

The following supporting information can be downloaded at https://www.mdpi.com/article/10.3390/diagnostics14242802/s1, Table S1: Names of features extracted from radiological images; Table S2: Optimal Hyperparameters of Machine Learning Models for Different Feature Selection Methods.
