# Supplementary material for: Diagnostic Models for Differentiating COVID-19-Related Acute Ischemic Stroke Using Machine Learning Methods
Source: Diagnostics (Basel). 2024 Dec 13;14(24):2802. doi: 10.3390/diagnostics14242802 (PMC11674536; doi:10.3390/diagnostics14242802)
Supplement: Supplementary file 1 [file diagnostics-14-02802-s001.zip › diagnostics-3254817-supplementary.pdf]

Supplementary Table 1: Names of features extracted from radiological images

**Feature Extraction**	**Feature Names**
Shape features (n=2)	diagnostics_Imageoriginal_Mean<br>diagnostics_Imageoriginal_Maximum
First Order Histogram features (n=18)	original_firstorder_10Percentile<br>original_firstorder_90Percentile<br>original_firstorder_Energy<br>original_firstorder_Entropy<br>original_firstorder_InterquartileRange<br>original_firstorder_Kurtosis<br>original_firstorder_Maximum<br>original_firstorder_MeanAbsoluteDeviation<br>original_firstorder_Mean<br>original_firstorder_Median<br>original_firstorder_Minimum<br>original_firstorder_Range<br>original_firstorder_RobustMeanAbsoluteDeviation<br>original_firstorder_RootMeanSquared<br>original_firstorder_Skewness<br>original_firstorder_TotalEnergy<br>original_firstorder_Uniformity<br>original_firstorder_Variance
Gray Level Co-occurrence Matrix (GLCM) features (n=24)	original_glcm_Autocorrelation<br>original_glcm_ClusterProminence<br>original_glcm_ClusterShade<br>original_glcm_ClusterTendency<br>original_glcm_Contrast<br>original_glcm_Correlation<br>original_glcm_DifferenceAverage<br>original_glcm_DifferenceEntropy<br>original_glcm_DifferenceVariance<br>original_glcm_Id<br>original_glcm_Idm<br>original_glcm_Idmn<br>original_glcm_Idn<br>original_glcm_Imc1<br>original_glcm_Imc2<br>original_glcm_InverseVariance<br>original_glcm_JointAverage<br>original_glcm_JointEnergy<br>original_glcm_JointEntropy<br>original_glcm_MCC<br>original_glcm_MaximumProbability<br>original_glcm_SumAverage<br>original_glcm_SumEntropy<br>original_glcm_SumSquares

Gray Level Difference Matrix (GLDM) features (n=14)	original_gldm_DependenceEntropy<br>original_gldm_DependenceNonUniformity<br>original_gldm_DependenceNonUniformityNormalized<br>original_gldm_DependenceVariance<br>original_gldm_GrayLevelNonUniformity<br>original_gldm_GrayLevelVariance<br>original_gldm_HighGrayLevelEmphasis<br>original_gldm_LargeDependenceEmphasis<br>original_gldm_LargeDependenceHighGrayLevelEmphasis<br>original_gldm_LargeDependenceLowGrayLevelEmphasis<br>original_gldm_LowGrayLevelEmphasis<br>original_gldm_SmallDependenceEmphasis<br>original_gldm_SmallDependenceHighGrayLevelEmphasis<br>original_gldm_SmallDependenceLowGrayLevelEmphasis
Gray Level Run Length Matrix (GLRLM) features (n=16)	original_glrlm_GrayLevelNonUniformity<br>original_glrlm_GrayLevelNonUniformityNormalized<br>original_glrlm_GrayLevelVariance<br>original_glrlm_HighGrayLevelRunEmphasis<br>original_glrlm_LongRunEmphasis<br>original_glrlm_LongRunHighGrayLevelEmphasis<br>original_glrlm_LongRunLowGrayLevelEmphasis<br>original_glrlm_LowGrayLevelRunEmphasis<br>original_glrlm_RunEntropy<br>original_glrlm_RunLengthNonUniformity<br>original_glrlm_RunLengthNonUniformityNormalized<br>original_glrlm_RunPercentage<br>original_glrlm_RunVariance<br>original_glrlm_ShortRunEmphasis<br>original_glrlm_ShortRunHighGrayLevelEmphasis<br>original_glrlm_ShortRunLowGrayLevelEmphasis
Gray Level Size Zone Matrix (GLSZM) features (n=16)	original_glszm_GrayLevelNonUniformity<br>original_glszm_GrayLevelNonUniformityNormalized<br>original_glszm_GrayLevelVariance<br>original_glszm_HighGrayLevelZoneEmphasis<br>original_glszm_LargeAreaEmphasis<br>original_glszm_LargeAreaHighGrayLevelEmphasis<br>original_glszm_LargeAreaLowGrayLevelEmphasis<br>original_glszm_LowGrayLevelZoneEmphasis<br>original_glszm_SizeZoneNonUniformity<br>original_glszm_SizeZoneNonUniformityNormalized<br>original_glszm_SmallAreaEmphasis<br>original_glszm_SmallAreaHighGrayLevelEmphasis<br>original_glszm_SmallAreaLowGrayLevelEmphasis<br>original_glszm_ZoneEntropy<br>original_glszm_ZonePercentage<br>original_glszm_ZoneVariance
Neighborhood Gray-tone Difference Matrix (NGTDM) features (n=5)	original_ngtdm_Busyness<br>original_ngtdm_Coarseness<br>original_ngtdm_Complexity<br>original_ngtdm_Contrast<br>original_ngtdm_Strength

Supplementary Table 2: Optimal Hyperparameters of Machine Learning Models for Different Feature Selection Methods

Model Name	Hyper-Parameter Name	Feature Selection Algorithms			
		NONE	BORUTA	LASSO	RFE
		Optimum value	Optimum value	Optimum value	Optimum value
k-NN	algorithm	auto	auto	auto	auto
	n_neighbors	5	5	3	3
	p	1	1	1	1
	weights	distance	distance	distance	Distance
SVM	C	10	10	10	10
	gamma	auto	scale	auto	Auto
	kernel	rbf	rbf	rbf	Rbf
RF	bootstrap	False	False	False	False
	max_depth	90	100	110	110
	max_features	2	3	3	3
	min_samples_leaf	3	3	3	3
	min_samples_split	10	10	8	8
	n_estimators	100	200	200	200
XGBoost	colsample_bytree	0.9	0.7	0.9	0.7
	learning_rate	0.1	0.1	0.1	0.1
	max_depth	5	5	5	5
	min_child_weight	1	3	1	5
	subsample	0.9	0.9	0.7	0.9
LR	C	10	1	0.1	10
	penalty	l2	l2	l2	l2
ANN	activation	tanh	relu	tanh	relu
	hidden_layer_sizes	(100.)	(100.)	(100.)	(200.)
